# Anti-Dengue ED3 Long-Term Immune Response With T-Cell Memory Generated Using Solubility Controlling Peptide Tags

**DOI:** 10.3389/fimmu.2020.00333

**Published:** 2020-03-17

**Authors:** Mohammad M. Islam, Shiho Miura, Mohammad N. Hasan, Nafsoon Rahman, Yutaka Kuroda

**Affiliations:** ^1^Department of Biotechnology and Life Science, Graduate School of Engineering, Tokyo University of Agriculture and Technology, Tokyo, Japan; ^2^Department of Biochemistry and Molecular Biology, University of Chittagong, Chittagong, Bangladesh

**Keywords:** solubility controlling peptide tags (SCP-tags), subvisible aggregates, dengue envelope protein domain 3 (ED3), immunogenicity, T-cell memory

## Abstract

Recombinant proteins are an attractive choice as a safe alternative to traditional live attenuated vaccines. However, most small-size proteins are poorly immunogenic, and adjuvants, whose mode of action remain to be fully clarified, are needed for increasing their immunogenicity. Here, we report the effects of short solubility controlling peptide tags (SCP-tags) on the immunogenicity of DENV3 envelope protein domain 3 (3ED3; 103 residues, 11.46 kDa) in ICR and Swiss albino model mice. The attachment of a 4-Ile SCP-tag (C4I-tag) increased the hydrodynamic radius of 3ED3 from 2.2 ± 0.09 to 111 ± 146 nm as assessed by dynamic light scattering in phosphate buffered saline at 37°C, indicating that the C4I-tag oligomerized 3ED3. Immunization at 30 μg/dose showed that the untagged 3ED3 was not or poorly immunogenic, whereas the C4I-tag increased its immunogenicity by up to 39-fold as assessed by the IgG level measured using ELISA. Moreover, the increased antibody level was sustained for over 6 months after immunization and a high number of effector and central memory T cells were generated. These observations provide solid and quantitative evidence for the hypothesis that subvisible aggregates with hydrodynamic radii of 100 nm can increase immunogenicity and that SCP-tag can establish a long-term, target-specific immune response in a way adequate for the development of a peptide/protein-based DENV vaccine.

## Introduction

Though recombinant proteins represent an attractive alternative to traditional live attenuated vaccines (1–3), they are poorly immunogenic (4, 5). Few protein-based vaccines have thus found a widespread usage, despite the simplicity of their production and handling (6). A protein's immunogenicity is influenced by an enormous number of intertwined factors, most of which are remotely controlled under standard biomedical practices, and rationales for controlling a protein's immunogenicity are lacking (7). Presently, adjuvants, which are known to increase immune response, are used both for clinical and biomedical purposes. However, the number of adjuvants approved for human vaccination is restricted (8, 9), and importantly, their mode of action remains unclear.

Beside the traditional adjuvants, protein aggregation is being reported to increase immune response (10–13). The aggregates can be prepared using various physical and chemical stresses, such as agitation or extreme pH (14). However, these processes are harsh and may affect the structural and biochemical integrity of the proteins and are avoided in biomedical practices (15, 16). More recently, fusion to hydrophobic/lipoprotein (15–17) and cytokines (18) have been reported to increase a protein's immunogenicity *in vitro* and *in vivo*, but here too their modes of action are not fully understood. A major reason for this lack of evidence is that techniques for controlling and monitoring the formation of submicron aggregates, without affecting the other biophysical and biochemical properties, are lacking.

We previously developed solubility controlling peptide tags (SCP-tags) for producing and controlling the formation of subvisible aggregates without compromising the structure, biochemical properties, and function of a model protein, a bovine pancreatic trypsin inhibitor (BPTI) variant (19–22). Here, we analyzed the effects of the SCP-tags on the aggregate's sizes of DENV3 envelope protein domain 3 (3ED3) and the anti-3ED3 immune response in model mice. ED3 is the third domain of the envelope glycoprotein, which constitutes the outermost layer of the dengue virion, and contains the epitope residues constituting the primary binding sites of neutralizing antibodies (23). We thus chose DENV3-ED3 (3ED3) as a good model for investigating the effects of subvisible aggregates of controlled sizes on the immunogenicity of 3ED3 in mice model. First, we show using dynamic light scattering (DLS) that 5-Lys (C5K), 5-Asp (C5D), and 4-Ile (C4I) tagged 3ED3 formed subvisible (soluble) aggregates with hydrodynamic radii of 2.9 ± 1.1, 78.5 ± 51.6, and 111 ± 146 nm, respectively. Next, immunization studies in model mice showed that C5K, C5D, and C4I tags increased the antibody titers in the order C4I > C5D > C5K, which was the same order as the aggregates hydrodynamic radii (C4I > C5D > C5K). Furthermore, the immunogenicity against 3ED3 was maintained for over 6 months, and a high level of effector and central memory T cells were produced. Altogether, our results suggest that SCP-tags could provide a versatile approach for increasing the immunogenicity of a protein through the manipulation of its aggregate's size, and as such, it may open the way for the development of protein-based vaccines.

## Materials and Methods

### Mutant Design

**Figure 1:**
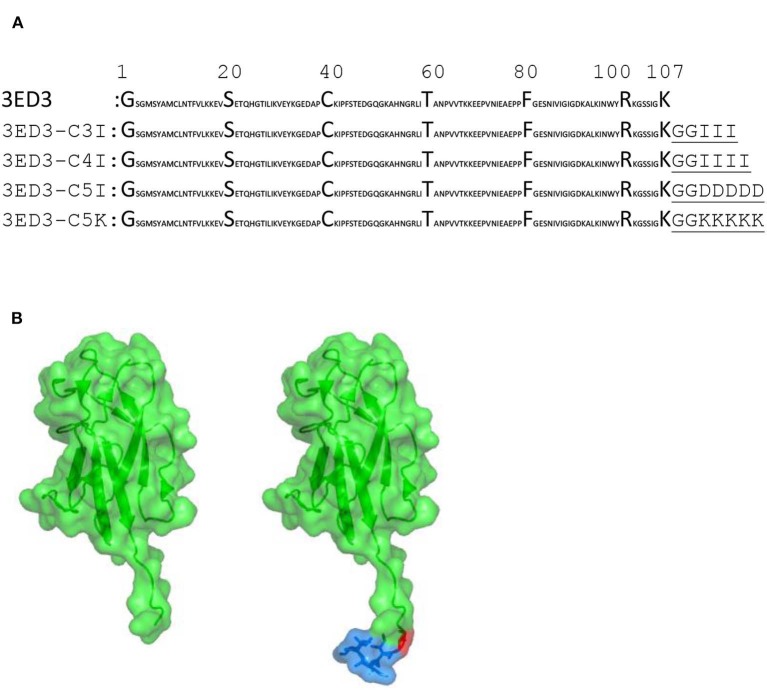
Sequences and structures of 3ED3 and its SCP-tagged variants. **(A)** The sequence of 3ED3 is taken from UniProt. The SCP-tag sequence, attached at the C termini of 3ED3, is underlined and consists of two glycine, used as a spacer, and 3, 4, or 5 amino acids. **(B)** Surface representation (green) of 3ED3 (left) and its SCP-tagged variant (right). The coordinates are taken from the PDB database 3VTT (24). The SCP-tag residues are in a surface-stick (blue) model. The model structures were generated using PyMOL (The PyMOL Molecular Graphics System, Version 2.0 Schrödinger, LLC).

The sequence of envelope protein domain 3 of dengue virus serotype 3 (3ED3) was retrieved from UniProt (ID P27915:1, residues 574–678). The gene encoding 3ED3 was artificially synthesized and cloned into a pET15b vector (Novagen) at the endonuclease NdeI and BamH1 sites, as previously described (24). The nucleotide sequences encoding the SCP-tags were added at the C terminus of 3ED3 by QuikChange (Stratagene, USA) site-directed mutagenesis, and all sequences were confirmed by DNA sequencing (Figure 1).

### Protein Expression and Purification

The 3ED3 variants were overexpressed in *E. coli* JM109(DE3)pLysS as inclusion bodies as reported earlier (25). After harvesting, the cells were lysed in lysis buffer (150 mM NaCl, 0.5% sodium deoxycholate, and 1% SDS in 50 mM Tris–HCl pH 8.5) and lysis wash buffer (lysis buffer supplemented with 1% *v*/*v* NP-40), and the cell lysates were air oxidized for 36 h at 30°C in 6 M guanidine hydrochloride in 50 mM Tris–HCl, pH 8.7. The His_6_-tagged 3ED3s were purified by Ni-NTA (Wako, Japan) chromatography, followed by dialysis against 10 mM Tris–HCl, pH 8.0 at 4°C. The N-terminal His_6_-tag was cleaved by thrombin proteolysis (25), and 3ED3s were purified by a second round of Ni-NTA chromatography followed by reversed-phase HPLC. Protein identities were confirmed by analytical HPLC and MALDI-TOF MS and stored at −30°C until use.

### Immunization Studies

A total of five sets of immunization experiments were carried out: four sets with Jcl:ICR (CLEA, Japan) and one set with Swiss albino (ICDDR,B, Bangladesh) mice, all aged 3–4 weeks at the start of the experiment. Four sets were carried out without adjuvant, and one set with ICR mice was carried out in the presence of Freund's adjuvant (26, 27). *Immunization in the presence of adjuvant*: the 3ED3 variants were dissolved in phosphate buffered saline (PBS), pH 7.4 at 30 μg/dose, and supplemented with an equal volume of Freund's adjuvants (100 μl protein plus 100 μl adjuvant; total 200 μl/dose/mice). The first dose was given subcutaneously in Freund's incomplete adjuvant, and doses 2–4 were given intraperitoneally at weekly intervals in Freund's complete adjuvant. *Immunization in the absence of adjuvant*: the 3ED3 variants were formulated in PBS, pH 7.4 at 30 μg/dose (100 μl/mice) and injected subcutaneously at weekly intervals unless otherwise specified. In addition, two control mice were injected with PBS and two with PBS–adjuvant at weekly intervals. Dose-specific immune response (anti-3ED3 IgM and IgG antibodies) was monitored 4 days after each inoculation using tail-bleed samples by ELISA. After the final dose, all mice were sacrificed, and blood samples were collected from the heart and centrifuged, and the sera were preserved at −30°C in aliquots of 50 μl until use. *Long-term immunogenicity*: we used a separate group of Jcl:ICR mice, and we inoculated the mice at 3-weeks intervals three times and monitored the serum's IgG levels weekly for over 6 weeks using ELISA. Similarly, IL-2 and IL-4 were measured 6 weeks after the last inoculation. *Flow cytometry*: in yet another group, Swiss albino mice were immunized at 2-weeks intervals, and after the final (fifth) dose, the anti-3ED3 IgG antibodies were monitored for over 6 months at weekly intervals by ELISA, and cell surface CD markers on splenocytes extracted 6 months after the last dose were investigated by flow cytometry. All of the experiments were performed in compliance with Tokyo University of Agriculture and Technology's and Japanese governmental regulations on animal experimentation.

### Immune Response Measured by ELISA

The effects of SCP-tags on the generation of 3ED3-specific serum antibody response in Jcl:ICR and Swiss albino mice models were investigated using ELISA in 96-well microtiter plates (Iwaki, Japan). The plates were coated overnight at room temperature with 2.5 μg/ml of purified untagged 3ED3 (unless otherwise specified) in PBS (100 μl/well). Unbound proteins were washed out, and the plates were blocked with 1% BSA in PBS for 45 min at 37°C. After washing with PBS, dose-specific mouse antisera were applied at 1:50 in 0.1% BSA in PBS, followed by a 3-fold serial dilution and incubated at 37°C for 2 h. Unbound antibodies were removed by thoroughly washing three times with PBS−0.05% Tween-20, and once with PBS. Microtiter plates were blot dried, and anti-mouse-IgG/IgM-HRP conjugates (Thermo Fisher Scientific; 1:3,000 dilution in 0.1% BSA–PBS−0.05% Tween-20) were added and incubated at 37°C for 90 min. The unbound conjugates were removed by washing three times with PBS−0.05% Tween-20 and once with PBS. Coloring was performed by adding the substrate OPD (*o*-phenylenediamine) at 0.4 mg/ml concentration supplemented with 4 mM H_2_O_2_ (100 μl/well). After 20 min of incubation at room temperature, the reaction was stopped by adding 50 μl of 1 N sulfuric acid, and the color intensity was measured at 492 nm (OD_492nm_) using a microplate reader (SH-9000Lab; Hitachi High-Tech Science, Tokyo, Japan). Antibody titers were calculated from the power fitting of OD_492nm_ vs. the reciprocal of the antisera dilution using a cutoff of OD_492nm_ = 0.1 above the background values. As a positive control, a previously developed anti-3ED3 sera (28) was used in all ELISA plates. The hydrodynamic radius of SCP-tagged 3ED3s was compared with the hydrodynamic radius of untagged 3ED3 using Dunnett's test (29). Similarly, multiple comparisons of the anti-3ED3 IgG antibody titers in treatment groups (mice injected with SCP-tagged 3ED3s) and control group (mice injected with untagged 3ED3) were performed using Dunnett's test (29). For data with *n* = 5 and above, values that were greater than the third quartile +1.5 × IQR or smaller than the first quartile −1.5 × IQR were considered as outliers.

### Cell Surface CD Marker Analysis

Single-cell suspension from spleen was prepared in FACS buffer (PBS supplemented with 2% FBS, 1 mM EDTA, and 0.1% sodium azide). The red blood cells (RBCs) were lysed with RBC lysis solution (0.15 M ammonium chloride, 10 mM potassium bicarbonate, 0.1 mM EDTA) for 5–10 min at room temperature, followed by washing twice with a FACS buffer (400 × *g*, 4°C, 5 min). Pellets (cells) were resuspended in a 100 μl pre-cooled FACS buffer. The cells were stained with anti-CD3-Pcy5, CD4-Pcy7, CD44-FITC, and CD62L-PE-conjugated antibodies in one tube and with anti-CD3-Pcy5, CD8-Pcy7, CD44-FITC, and CD62L-PE-conjugated antibodies in another tube (0.2 μg of antibodies/100 μl) for 30 min in the dark. Excess unbound conjugated antibodies were removed by washing the cells with a FACS buffer. Finally, cells were resuspended in a 500 μl FACS buffer, and the data were collected using CytoFlex (Beckman Coulter). The levels of IL-2 and IL-4 in the serum from the heart-bleed samples collected after the final dose of immunization were measured by ELISA following the manufacturer's (BioLegend) protocol.

### Dynamic Light Scattering

**Figure 2:**
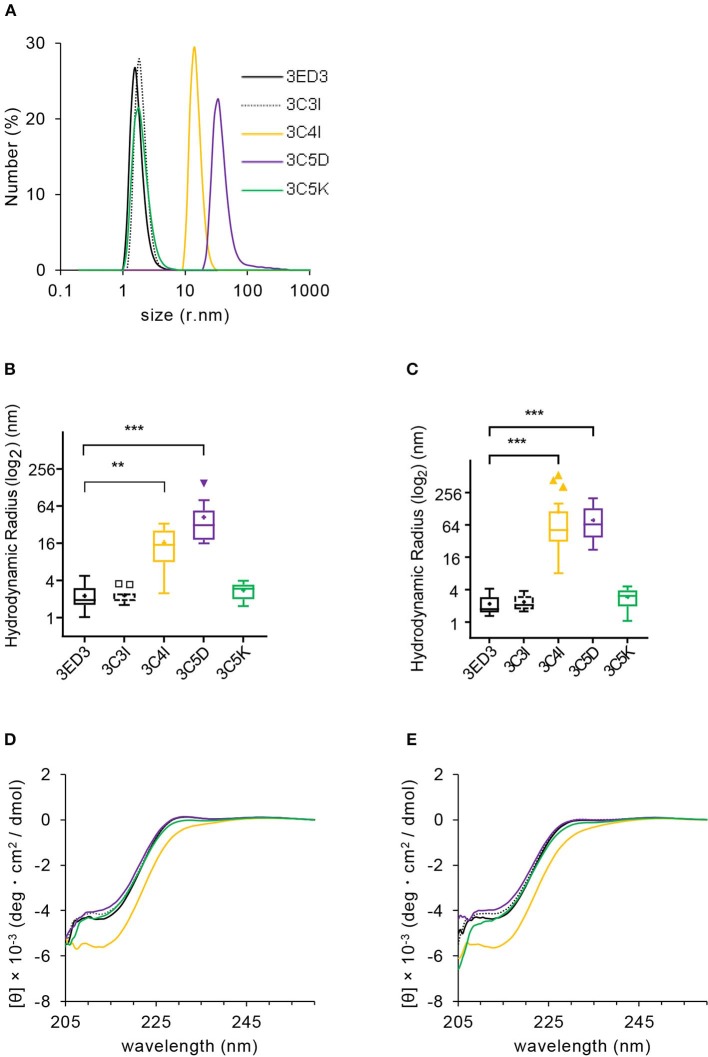
Biophysical characterization of 3ED3 and its SCP-tagged variants. **(A)** Hydrodynamic radii of 3ED3 aggregates as computed from the number (%) distribution measured at 0.3 mg/ml protein concentration in PBS, pH 7.4 at 25°C. Hydrodynamic radius of 3ED3 and its SCP-tagged variants at 25°C **(B)** and 37°C **(C)** in the immunization samples monitored just before inoculation to the ICR mice (100 μl at 0.3 mg/ml in PBS, pH 7.4; titers are reported in Figures 3, 4A). Asterisks represent the comparisons using Dunnett's test (28) [**(B)** *n* = 29 (3ED3), 11 (3C3I), 20 (3C4I), 17 (3C5D), 18 (3C5K); **(C)** *n* = 36 (3ED3), 14 (3C3I), 20 (3C4I), 18 (3C5D), 19 (3C5K); “+”: mean, **p < 0.01, ***p < 0.001]. Secondary structure of 3ED3 variants measured by CD at 25°C **(D)** and 37°C **(E)** at 0.3 mg/ml in PBS, pH 7.4. Color codes are the same in all panels and are shown in **(A)**.

**Figure 3:**
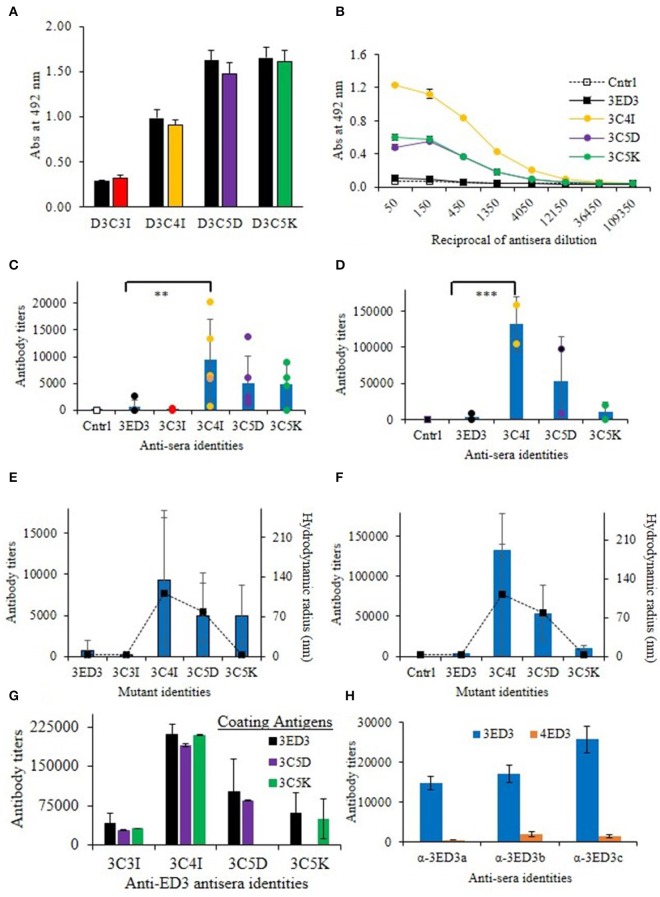
Immunogenicity of 3ED3 and its SCP-tagged variants. **(A)** Anti-3ED3 specificity of antisera generated against 3ED3 and its SCP-tagged variants assessed by ELISA. OD_492nm_ at 1:50-fold antisera dilution is shown (black: against 3ED3; red: against 3C3I; orange: against 3C4I; violet: against 3C5D; green: against 3C5K as coating antigen). **(B)** Absorbance at 492 nm vs. the reciprocal of antisera dilution against 3ED3 (legends are given in the panel). **(C)** IgG antibody titer after the fourth dose in the absence of the adjuvant [*n* = 2 (PBS), 9 (D3wt), 2 (D3C3I), 5 (D3C4I), 5 (D3C5D), 4 (D3C5K); **P < 0.05, ***P < 0.001 (Dunnett's test) (29)]. **(D)** IgG antibody titers after the fourth dose in the presence of the adjuvant. Immunization experiments were carried out in the Jcl:ICR (CLEA, Japan) mice model. Bars represent averaged antibody titer, and scattered points stand for individual mice data [color codes are as in **(B)**]. Correlation between the antibody titers (bars on the primary axis) and the aggregates' hydrodynamic radii at 37°C in the absence of the adjuvant **(E)** and in the presence of the adjuvant **(F)**. Scattered points along the secondary axis stand for hydrodynamic radii. **(G)** Antibody titers determined against untagged (3ED3) and its SCP-tagged (C5D and C5K) variants. **(H)** ELISA data for anti-3ED3s sera against 3ED3 and 4ED3. All anti-3ED3 sera showed sero-specific recognition 3ED3 and no cross-recognition of 4ED3, confirming that all antibodies were serotype specific.

**Figure 4:**
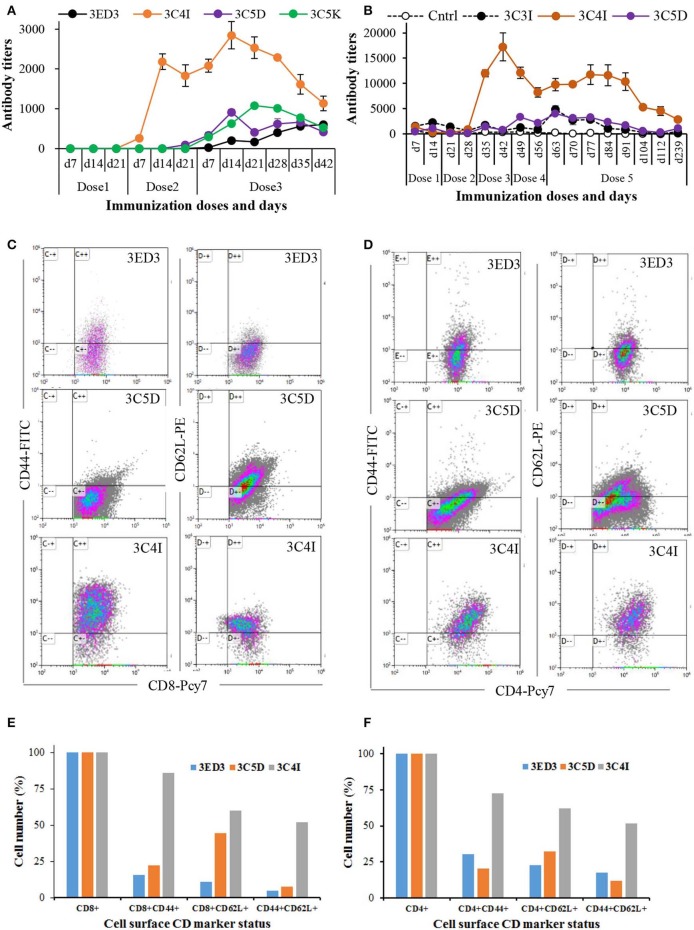
Long-term immune response and surface CD marker analysis. The immunization was carried out at 3-weeks intervals in Jcl:ICR mice **(A)** and at 2-weeks intervals in Swiss albino mice **(B)**. Immunization experiments were carried out in the absence of adjuvants (100 μl at 0.3 mg/ml in PBS, pH 7.4). After the final dose, mice were monitored for 6 weeks **(A)** and 6 months **(B)** by measuring the anti-3ED3 IgG level by ELISA. Mice with high antibody titers are shown (the data for the other mice are given in Supplementary Figure S4). SCP-tagged induced differential expression of surface CD markers on Tc cells **(C)** and on Th cell **(D)** are shown (identities of 3ED3 variants are shown at the top of dot plots). Mice injected with C4I-tagged 3ED3 had very high population of CD44^+^ and CD44^+^CD62L^+^ Tc cells and Th cells, respectively. Differential expression of CD44 and CD62L on Tc cells and Th cells are shown in **(E)** and **(F)**. C4I-tagged 3ED3 induced both T-cell effector and memory function, ensuring long-term immune responses.

The effects of SCP-tags on subvisible aggregates' sizes were investigated using a Malvern Zetasizer Nano-S system (Malvern, UK) in PBS, pH 7.4. Samples were prepared in PBS at 0.3 and 0.8 mg/ml concentration, kept on ice, and centrifuged (20,000 × *g* at 20°C for 20 min) just before DLS measurements. Then 100 μl of supernatants was transferred into a cuvette, and DLS measurements were conducted at 4, 25, and 37°C. The hydrodynamic radius (*R*_h_) was calculated from the number and volume distribution using the Stokes–Einstein equation, and the mean *R*_h_ was calculated from three or more independent experiments (30). In addition, we monitored the aggregates' sizes of the 3ED3 variants directly from the injection samples, before each inoculation to ICR mice (reported in Figures 2–4).

### Thermal Stability by Circular Dichroism (CD)

The effects of C4I, C5D, and C5K tags on structure conformation were monitored by CD by dissolving the lyophilized protein powders in PBS, pH 7.4. Before CD measurements, all samples were centrifuged at 20,000 × *g* for 20 min at 4°C to remove aggregates that might have accumulated during sample preparation. The CD spectra were measured using a 2-mm cuvette with a JASCO J-820 spectropolarimeter at 0.10–0.45 mg/ml concentration in the temperature range 20–90°C and in the wavelength range 200–260 nm. The reversibility of thermal unfolding–refolding was confirmed by measuring the CD of the sample after cooling it back to 20°C from 90°C.

## Results and Discussion

### Biophysical Properties of Subvisible Aggregates

We first analyzed the biophysical properties of the 3ED3 variants under conditions identical or very close to that used in the immune response experiment (at 0.3 mg/ml protein concentration in PBS at 25 and 37°C). Overall, the results were in line with those expected from our previous studies, where we used a BPTI variant to assess the effect of the SCP-tags on the protein's structure, oligomerization status, and thermodynamic stability (19–22). Namely, all 3ED3 variants were “soluble” as they remained in the supernatant after centrifugation at 20,000 × *g* for 20 min at 25°C, or after filtration through a 0.22-μm membrane filter (Figure 2; Supplementary Figure S1). Thus, they either were monomeric or formed very small oligomeric subvisible particles (31).

We thus characterized the sizes of the subvisible aggregates and their dependence on protein concentration by DLS at 25 and 37°C at 0.3 mg/ml, which was the inoculation concentration, and at 0.8 mg/ml (Figures 2A–C, Supplementary Figures S1A–D). The particle size of the wild-type 3ED3 was almost independent of both the sample's temperature and the protein's concentration (Supplementary Figures S1C,D). C5K and C3I did not affect the aggregate sizes, whereas the C4I and C5D dramatically increased the subvisible aggregate sizes to *R*_h_ = 111 ± 146 and 78 ± 51 nm, respectively (Figures 2A–C). The probable molecular origins for the increase in particle size are discussed in our previous studies with BPTI (32). Namely, C5D-tag most likely oligomerized the protein through electrostatic interactions between the negatively charged side chains of C5D tag with positive charges on 3ED3's surface. On the other hand, C4I-tag acts through intermolecular hydrophobic interactions between the Ile attached to the 3ED3 molecules inducing a reversed hydrophobic effect (33). Prolonged incubation at 25 and 37°C under the immunization conditions did not change the aggregate sizes of the 3ED3 variants, except that of the C4I variant, whose size increased gradually over time (Supplementary Figure S1E). Finally, CD spectra at 0.3 mg/ml protein concentration in PBS, pH 7.4 indicated that at 37°C all 3ED3 variants retained the same secondary structures, but the C4I variant was slightly destabilized, as reported for BPTI (32) (Figures 2D,E; Supplementary Figure S2). Altogether, these observations indicated different subvisible aggregation mechanisms and different aggregate sizes for C5D and C4I-tagged 3ED3s.

### Effects of SCP-Tags on Serum Antibody Response in Model Mice

We investigated the effects of SCP-tags on the generation of 3ED3-specific serum antibody response through repeated injections in ICR mice, both in the presence and absence of Freund's adjuvant. Noteworthy, we measured the 3ED3s' hydrodynamic radii directly in the immunization sample prior to each round of inoculation in order to monitor the sample's oligomerization status in a “real-time” manner (Figures 2A–C; Supplementary Figure S2). Let us also note that the OD_492nm_ of the anti-3ED3 sera measured using plates coated with the untagged 3ED3 and 4ED3 and SCP-tagged 3ED3s were essentially the same, indicating that the antibodies were 3ED3 specific (sero-specific) (Figure 3H) and were directed against the 3ED3 scaffold, not against the SCP-tags (Figures 3A,G; Supplementary Figure S5A).

First, let us consider the immunization without the adjuvant. The untagged 3ED3 was not or very poorly immunogenic even after the fourth dose (Figures 3C,D; Supplementary Figure S3), whereas the immunogenicity of C5K, C5D, and C4I appeared after the third dose. After the fourth dose, the respective titer increased to 13, 20, and 30 times that of the untagged 3ED3 (Figure 3C; Supplementary Figure S3). Noteworthy, 3ED3-C3I (3C3I), whose SCP-tag is shorter than that of 3ED3-C4I (3C4I) by merely a single isoleucine, was poorly immunogenic, which was in line with the fact that C3I-tag did not oligomerize 3ED3 (Figures 2C, 3E). However, two out of four mice injected with 3C5K exhibited antibody titers >5,000 (Figure 3C) showing an exception to the otherwise good correlation between the aggregate sizes and immunogenicity, but currently, we do not have a good molecular level explanation for these two cases.

Similar trends were observed in immunization experiments in the presence of Freund's adjuvant (Figure 3D). Namely, after the fourth dose, C5K, C5D, and C4I increased antibody titers up to 5-, 24-, and 39-folds, respectively, over the untagged 3ED3, indicating that C4I-tagged 3ED3 was the most immunogenic followed by C5D, and C5K and the untagged 3ED3 remained non-immunogenic (Figure 3D; Supplementary Figure S3). Note that the immune response was specific to DEN3-ED3 as no IgG against DEN4-ED3 was observed by ELISA (Figure 3H). Finally, very similar trends of increased immune responses were observed both in the presence and absence of the adjuvant. This strongly suggests that the SCP-tags are the sole contributor for increasing the immunogenicity of the poorly immunogenic 3ED3.

### Long-Term Antibody Response and Cytokine Expression

The effects of the SCP-tags on long-term immune response were monitored by measuring the serum antibody titers for 6 weeks to 6 months after the last dose of immunization (Figure 4; Supplementary Figure S4). The anti-3ED3 antibody titers produced by the C4I-tagged variant remained very high for 5–6 weeks (Figures 4A,B). Even after 6 months, C4I-injected mice had anti-3ED3 antibody levels over three times higher than those observed with other variants. These observations clearly indicated that a long-term anti-3ED3 immune response was established in mice inoculated with 3ED3-C4I (Figures 4A,B; Supplementary Figures S4A,B).

To assess the potential of the tagged 3ED3s as anti-dengue 3 vaccine candidates, we performed a CD marker analysis. The levels of CD markers on lymphocytes 6 months after immunization indicated that the untagged 3ED3 and its C5D-tagged variants had a low number of CD44^+^ and CD62L^+^ Tc and Th cells (Figures 4C–F). To be more precise, only 5–44 and 12–32% of Tc and Th cells, respectively, were CD44^+^, CD62L^+^, and CD44^+^CD62L^+^ in mice injected with untagged 3ED3 and its C5D-tagged variant (Figures 4E,F), indicating their naïve immunological status (34, 35). On the other hand, C4I-injected mice had a very high number of CD44^+^ Tc cells (86% of Tc cells) with increased expression of CD62L (on 60% of Tc cells) and co-expression of CD44-CD62L (CD44^+^CD62L^+^, on 52% of Tc cells) as well (Figures 4C,E), which is an indication of long-term immunity with effector Tc-cell memory (36). Furthermore, a very high population of CD44^+^, CD62L^+^, and CD44^+^CD62L^+^ Th cells (on 72, 62, and 52% of Th cells, respectively) (Figures 4D,F) suggested that repeated injections of C4I-tagged 3ED3 also developed central Th-cell memory (37). Such high expression of CD44 on T cells may seem unusual, but not rare (34–40). For example, an aerosol infection of *Mycobacterium tuberculosis* in mice resulted a drastic shifting of CD44 and CD62L expression on T cells from 18–32% to 59–79% and from 9–20% to 40–73%, respectively (41), similar to what we observed in this study. Similarly, choriomeningitis virus infection studies in mice also reported long-term Th-cell memory with high expression of CD44 on T cells (42). In addition, the total number of CD4- and CD8-positive cells remained very similar in all mice groups (untagged, C5D-tagged, and C4I-tagged ED3-injected mice groups) (Supplementary Figure S6), indicating that the increased number of activated CD4/CD8 cells (with high CD44 expression) in the spleen represents an expansion of preliminary sensitized T cells, rather than the recruitment of new T cells in spleen following ED3 injection (42). CD44 is a cell surface adhesion receptor and its ligation augments T-cell activation, survival, differentiation, and maintenance of memory (38). In addition, CD44 is widely used as a marker for antigen-encountered, effector, and memory T cells and thus for distinguishing memory T cells from naïve T cells (38, 39). Thus, the high and long-lasting IgG responses along with the high expression of CD44 and CD62L on T cells, which was observed only with the C4I variant, clearly indicated that the C4I tag oligomerized 3ED3 into subvisible (nanometer scale) particles, possibly enabling an efficient uptake of the antigen by antigen presenting cells (14), which in turn supposedly enhanced T-cell activation and antibody production (40).

Altogether, these observations indicated that the T cells in mice injected with the untagged or the C5D-tagged 3ED3 remained naïve or in an early stage of effector T-cell phenotype (43). On the other hand, C4I-tagged 3ED3 turned naïve or primarily sensitized effector T cells into effector/memory cells (44). These observations together with the long-lasting anti-3ED3 IgG antibody response strongly suggested that C4I-tagged 3ED3 did generate an adequate effector and central T-cell memory (Figure 4; Supplementary Figure S4), which was further corroborated by the increased expression of IL-4 (Supplementary Figure S4C). Thus, although a detailed study of the mechanism of the C4I-mediated conversion of naïve T cells into effector/memory phenotype may be needed in order to fully confirm a direct link between the state of the T cell and the long-term memory responses, both the observation of the long-term immunity and the CD marker analysis suggest the strong potential of C4I as a basic technology for developing efficient protein/peptide-based vaccines (45, 46).

### Immune Response and Aggregate Sizes

SCP-tag is a versatile method for controlling the formation of soluble aggregates, and as such, it can provide an avenue for a protein-based vaccine, as discussed above. In addition, SPC's ability to reliably control the formation of aggregates can contribute to shed light on the effects of aggregation on immune response, which remains to be fully clarified. We thus conducted all biophysical measurements (DLS, SLS, CD, etc.) under conditions as close as possible to that used in the immunization experiment (0.3 mg/ml concentration in PBS, pH 7.4 at 37°C); moreover, we monitored the aggregates' sizes of the inoculation samples, in a “real time” manner, i.e., just before injection to the ICR mice using an aliquot of the injected samples. We believe that the latter precaution is critical because aggregates can form in a little predictable manner upon small variation of external factors, such as room temperature or during freeze–thaw. Thus this “real time” monitoring substantially increased the reliability of our observation, thereby facilitating the analysis of the effects of subvisible aggregates on the immune response (Figures 2, 3).

The SCP-tags increased the aggregation sizes according to the following order: C4I > C5D > C5K > C3I > 3ED3 (Figure 2), which is the same order as the immunogenicity increase measured by anti-3ED3 IgG antibody titers (Figure 3); the C4I variant formed the largest subvisible aggregates and was the most immunogenic variant (Figure 3). Note that C5D and C4I tagged 3ED3, which formed subvisible aggregates of, respectively, 78 and 111 nm, were stabilized by different types of interactions: electrostatic interaction for C5D and hydrophobic interactions for C5I (Figures 3D,E). Thus, altogether, we observed a strong correlation between the hydrodynamic radius and the immunogenicity (Figure 3F). With one exception being 3C5K in the absence of the adjuvant, the molecular mechanism for this discrepancy remains to be fully explored.

## Conclusions

The prime goal of our study was not vaccine development against a specific virus but rather to propose a novel strategy for designing potent peptide/protein-based vaccines. Here, we have shown that the formation of subvisible protein aggregates with sizes ranging from a few tens-to-hundred nanometers can be produced using SCP-tags and that the appearance of aggregates correlates with the increased immunogenicity of 3ED3. The biophysical and biochemical nature of subvisible protein aggregates smaller than 1 μm has barely been characterized, and their physiological effects have often been overlooked (47, 48). This is thus the first report demonstrating such a strong correlation between the size of the aggregates and the strength of the immune response with a near-real-time monitoring of the aggregates. Furthermore, cytokine analysis indicated that the C4I-tagged 3ED3 induced a long-lasting anti-3ED3 IgG antibody response with adequate effector and central T-cell memory. Altogether, SCP-tags appear to be a versatile technique for increasing the immunogenicity of a protein in a targeted manner, and provided the neutralization assay is successful, it could provide a new approach for developing efficient protein and peptide-based vaccines (34, 35).

## Data Availability Statement

All datasets generated for this study are included in the article/Supplementary Material.

## Ethics Statement

The animal study was reviewed and approved by TUAT animal experimentation ethics committee.

## Author Contributions

YK and MI designed the project and wrote the manuscript. MI and SM performed the experiments and analyzed the data. MH conducted the long-term immune response study and flow cytometry analysis, and NR helped with experimentation. All authors read and approved the manuscript.

### Conflict of Interest

The authors declare that the research was conducted in the absence of any commercial or financial relationships that could be construed as a potential conflict of interest.
