# Supplementary material for: Anti-Dengue ED3 Long-Term Immune Response With T-Cell Memory Generated Using Solubility Controlling Peptide Tags
Source: Front Immunol. 2020 Mar 17;11:333. doi: 10.3389/fimmu.2020.00333 (PMC7089932; doi:10.3389/fimmu.2020.00333)
Supplement: Supplementary file 1 [file Data_Sheet_1.pdf]

Supplemental Information

Anti-Dengue ED3 long-term immune response with T-cell memory generated using Solubility Controlling Peptide tags

Mohammad M. Islam[1,2], Shiho Miura[2], Mohammad. N. Hasan[1], Nafsoon Rahman[2], Yutaka Kuroda[2*]

[1]Department of Biochemistry and Molecular Biology, University of Chittagong, Chittagong 4331, Bangladesh; [2]Department of Biotechnology and Life Science, Graduate School of Engineering, Tokyo University of Agriculture and Technology, 2-24-16 Nakamachi, Koganei-shi, Tokyo 184-8588, Japan.

Figure legends

Suppl. Fig. S1. Biophysical characteristics of subvisible protein aggregates. (A) Effects of filtration through 0.22μm membrane filter. A indicates after filtration and B before filtration. (B) effects of centrifugation at 20000xg for 20min. C stands for after centrifugation and F after filtration. (C) effects of temperature, and (D) effects of protein concentration on subvisible protein aggregate sizes of untagged 3ED3 and its SCP-tagged variants. The subvisible aggregates of nanometer dimensions were fully soluble. (E) Effects of prolonged incubation time on subvisible aggregate sizes. Incubation temperature was 25, 37 and 25^{0}C (for reversibility checking) for 0-25 minutes, 26-55 minutes, and 56-80 minutes, respectively. Aggregate sizes for 3ED3, and its C3I, C5D and C5K tagged variants remained almost constant over time and temperature. However, the aggregate sizes increased over time and temperature for C4I tagged variant. (F) Biophysical stability (CD spectra) of untagged 3ED3 its SCP-tagged variants at 37^{0}C. All 3ED3 variants had very similar CD spectra. Legends are shown within the panels. CD spectra at 0.3mg/mL protein concentration in PBS, pH7.4, under the DLS condition, indicated that at 37°C all 3ED3 variants retained the same secondary structures. D3 stands for 3ED3.

Suppl. Fig. S2. Concentration dependent CD-spectra of 3ED3 and its SCP-tagged variants. CD spectra were measured at 0.1-0.45mg/mL protein concentration in PBS (pH7.4) at 37^{0}C. CD spectra of untagged 3ED3 (A), C5D (B), C5K (C) and C4I (D) tagged 3ED3 are shown. With increasing protein concentration extent of destabilization increased and decreased for C5D and C4I variants, respectively. On the otherr hand, the CD spectra for 3ED3 and its C3I variant was independent of protein concentration. Such decrease in stability of C4I variant could be originated from reverse hydrophobic effects[31] of the hydrophobic tag residues in their oligomeric state as discussed previously.

Suppl. Fig. S3. Dose-dependent serum anti-3ED3 antibody responses. Dose-specific antibody responses in PBS (in the absence of adjuvant) are shown in A and B (A: experiment 1; B: experiment 2). (C) Dose-specific IgG antibody response in the presence of Freund's adjuvant. The patterns of SCP-tag-dependent antibody titers remained almost the same both in the presence and absence of adjuvant. Immune response appeared after the 3rd dose. Interestingly, increased in anti-3ED3 IgG antibody titers went over 13, 20 and 30-folds with C5K, C5D and C4I, respectively after 4th dose (in the absence of adjuvant), while in the presence of adjuvant it

was over 5, 24 and 39 fold, respectively for C5K, C5D and C4I tagged 3ED3s. (D) Anti-3ED3 antibody response after 4th dose (at two weeks intervals) in Swiss albino mice in the absence of adjuvant. (E) Anti-3ED3 antibody response after 3rd dose (at three weeks intervals) in Jcl:ICR mice in the absence of adjuvant. In panels D and E, bars represent averaged data while asterisks (*) stand for individual mice data. All experiments were independent and conducted at different times with different protein lots. (F) Dose-specific anti-3ED3 IgM antibody titers. Repeated immunization at weekly intervals was conducted in the presence of adjuvant in Jcl:ICR mice.

Suppl. Fig. S4. Effects of SCP-tags on long-term antibody response in Jcl:ICR and Swiss albino mice model. Doses at two weeks intervals in Swiss albino mice (A), and at three weeks intervals in Jcl:ICR mice (B). Immunization experiments were carried out in the absence of adjuvants, and after the final dose Anti 3ED3 IgG were monitored for over 6 weeks (ICR; B) and 6 months (Swiss Albino; A) by ELISA. Averages of at least two mice in each set (for each variant) are shown. C4I generated the highest long-lasting IgG antibodies. (C) Serum IL-2 and IL-4 levels in mice immunized with 3ED3 and its SCP-tagged variants measured in serum after last dose of immunization. Mice immunized with C4I had low IL-2 and slightly high IL-4 in their sera, indicating that C4I induced a Th2-dependent humoral immune response against 3ED3.

Suppl. Fig. S5. Specificity of anti-3ED3 antibodies (anti-sera). (A) ELISA data for untagged and SCP-tagged 3ED3.

Suppl. Fig. S6. SCP-tag induced induced differential expression of surface CD markers on T cells. Expression of CD44 and CD62l on Th and Tc cells are shown in panel A and B, respectively. Mice injected with C4I-tagged 3ED3 had very high population of CD44+ and CD44+CD62L+ Tc-cell and Th-cells, respectively. In all cases (untagged, C5D-tagged and C4I-tagged ED3 injected mice), the overall number of total CD4 and CD8 positive cells remained very similar, indicating that the increased number of activated CD4/CD8 cells (with high CD44 expression) in the spleen represents the expansion of preliminary sensitized T cells, rather that the recruitment of new T cells in the spleen.

Suppl. Fig. S1.

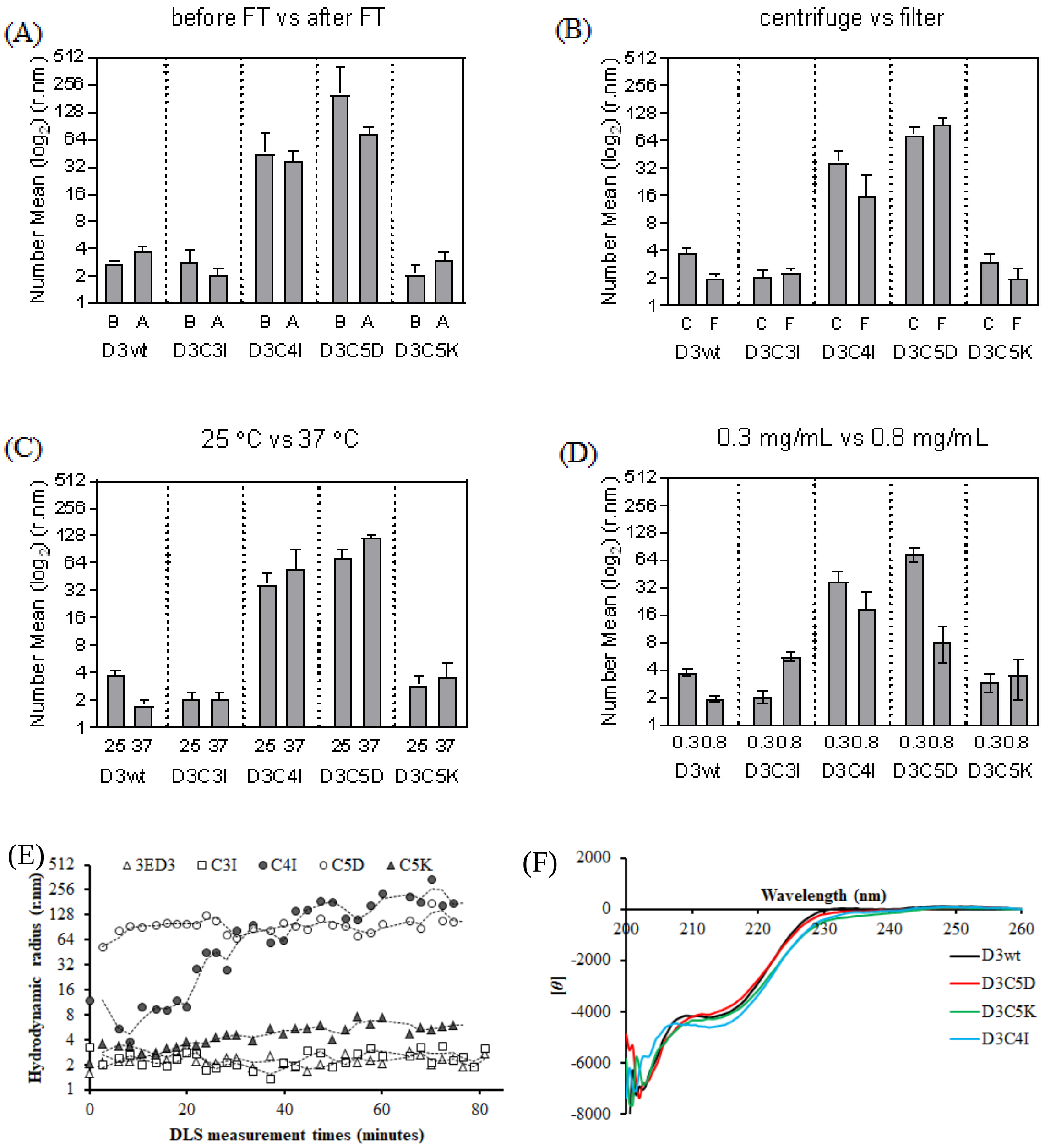
(A)
before FT vs after FT
Number Mean (log2) (r.nm)
512
256
128
64
32
16
8
4
2
1
B A B A B A B A B A
D3wt D3C3I D3C4I D3C5D D3C5K
(B)
centrifuge vs filter
Number Mean (log2) (r.nm)
C F C F C F C F C F
D3wt D3C3I D3C4I D3C5D D3C5K
(C)
25 °C vs 37 °C
Number Mean (log2) (r.nm)
25 37 25 37 25 37 25 37 25 37
D3wt D3C3I D3C4I D3C5D D3C5K
(D)
0.3 mg/mL vs 0.8 mg/mL
Number Mean (log2) (r.nm)
0.3 0.8 0.3 0.8 0.3 0.8 0.3 0.8 0.3 0.8
D3wt D3C3I D3C4I D3C5D D3C5K
(E)
3ED3 C3I C4I C5D C5K
Hydrodynamic radius (r.nm)
0 20 40 60 80
DLS measurement times (minutes)
(F)
2000
0
-2000
-4000
-6000
-8000
Wavelength (nm)
200 210 220 230 240 250 260
[θ]
D3wt
D3C5D
D3C5K
D3C4I

Suppl. Fig. S2.

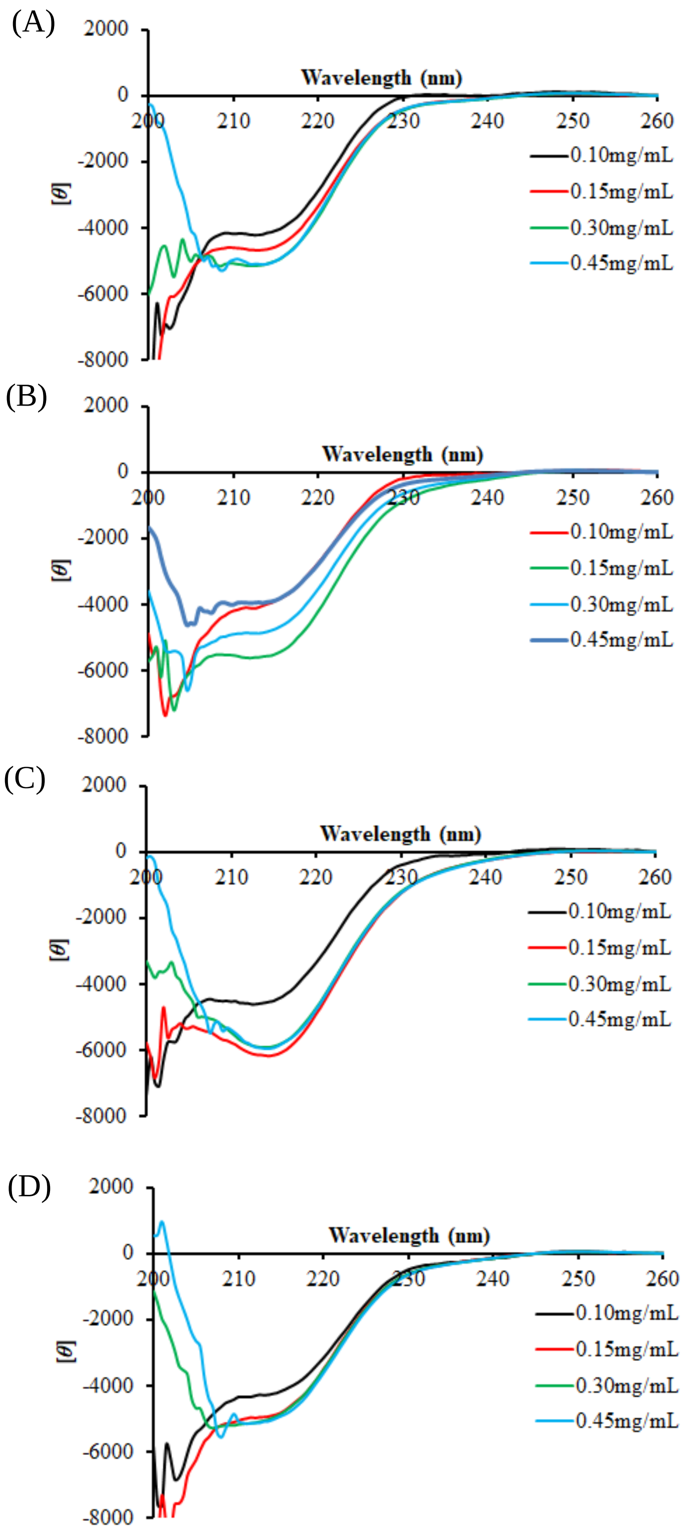
(A)
Wavelength (nm)
[θ]
2000
0
-2000
-4000
-6000
-8000
200
210
220
230
240
250
260
0.10mg/mL
0.15mg/mL
0.30mg/mL
0.45mg/mL
(B)
Wavelength (nm)
[θ]
0.10mg/mL
0.15mg/mL
0.30mg/mL
0.45mg/mL
(C)
Wavelength (nm)
[θ]
0.10mg/mL
0.15mg/mL
0.30mg/mL
0.45mg/mL
(D)
Wavelength (nm)
[θ]
0.10mg/mL
0.15mg/mL
0.30mg/mL
0.45mg/mL

Suppl. Fig. S3.

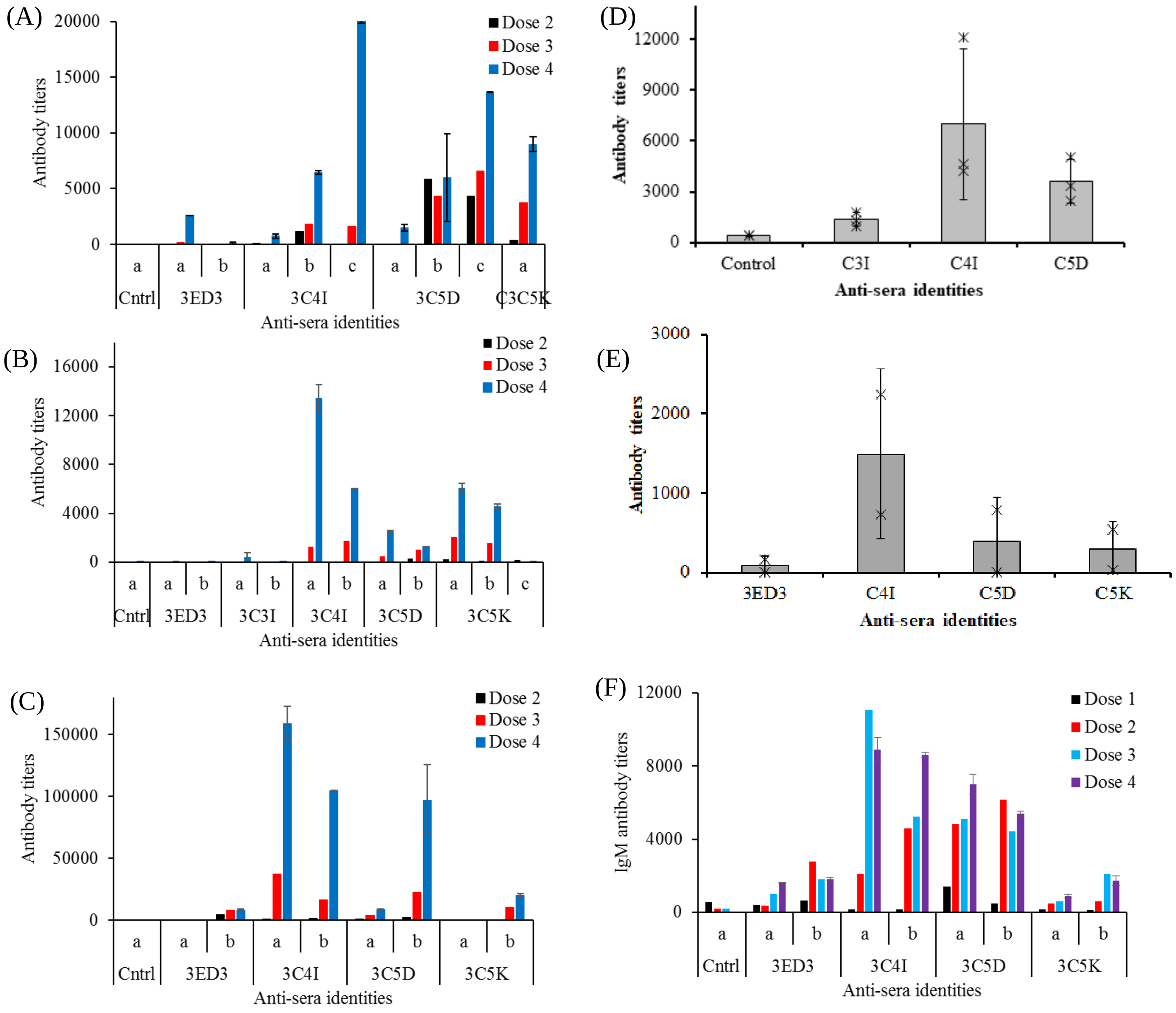
(A)
Antibody titers
20000
15000
10000
5000
0
Dose 2
Dose 3
Dose 4
a a b a b c a b c a
Cntrl 3ED3 3C4I 3C5D C3C5K
Anti-sera identities
(B)
Antibody titers
16000
12000
8000
4000
0
Dose 2
Dose 3
Dose 4
a a b a b a b a b a b c
Cntrl 3ED3 3C3I 3C4I 3C5D 3C5K
Anti-sera identities
(C)
Antibody titers
150000
100000
50000
0
Dose 2
Dose 3
Dose 4
a a b a b a b a b
Cntrl 3ED3 3C4I 3C5D 3C5K
Anti-sera identities
(D)
Antibody titers
12000
9000
6000
3000
0
Control C3I C4I C5D
Anti-sera identities
(E)
Antibody titers
3000
2000
1000
0
3ED3 C4I C5D C5K
Anti-sera identities
(F)
IgM antibody titers
12000
8000
4000
0
Dose 1
Dose 2
Dose 3
Dose 4
a a b a b a b a b
Cntrl 3ED3 3C4I 3C5D 3C5K
Anti-sera identities

Suppl. Fig. S4.

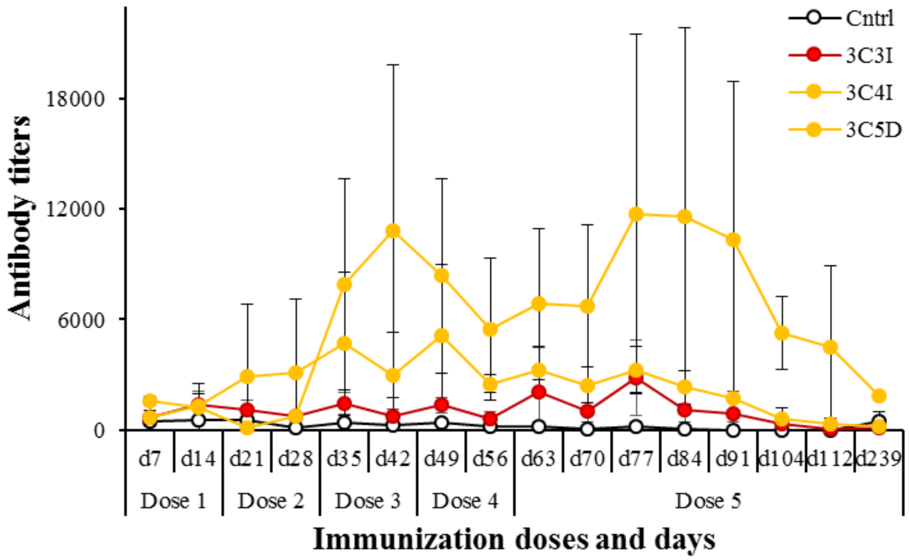

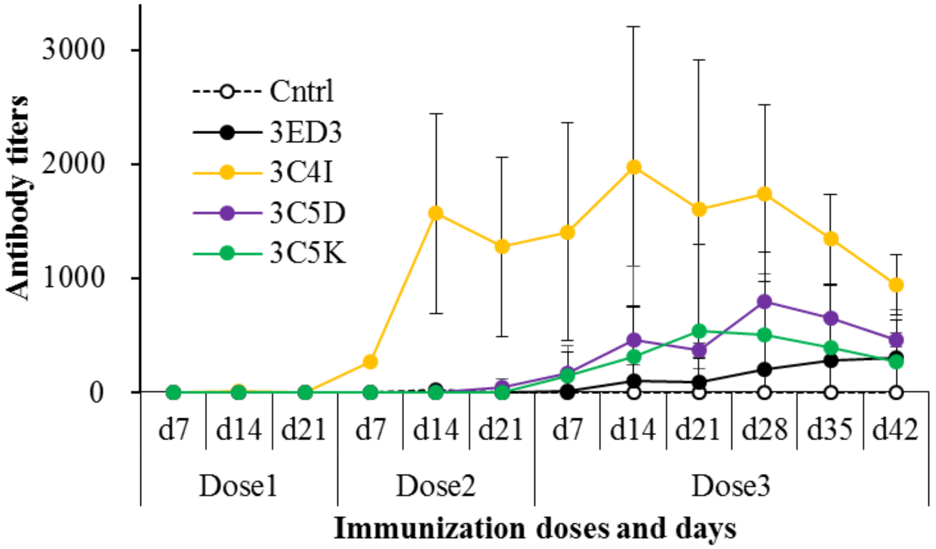

(C)

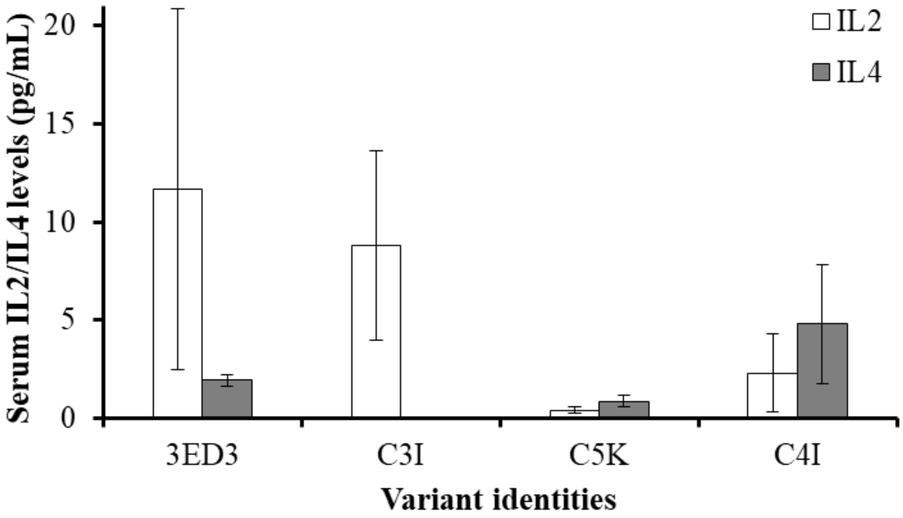

Suppl. Fig. S5.

(A)

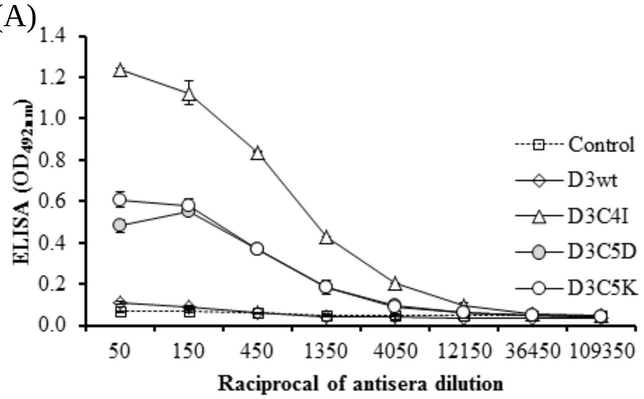

Suppl. Fig. S6.

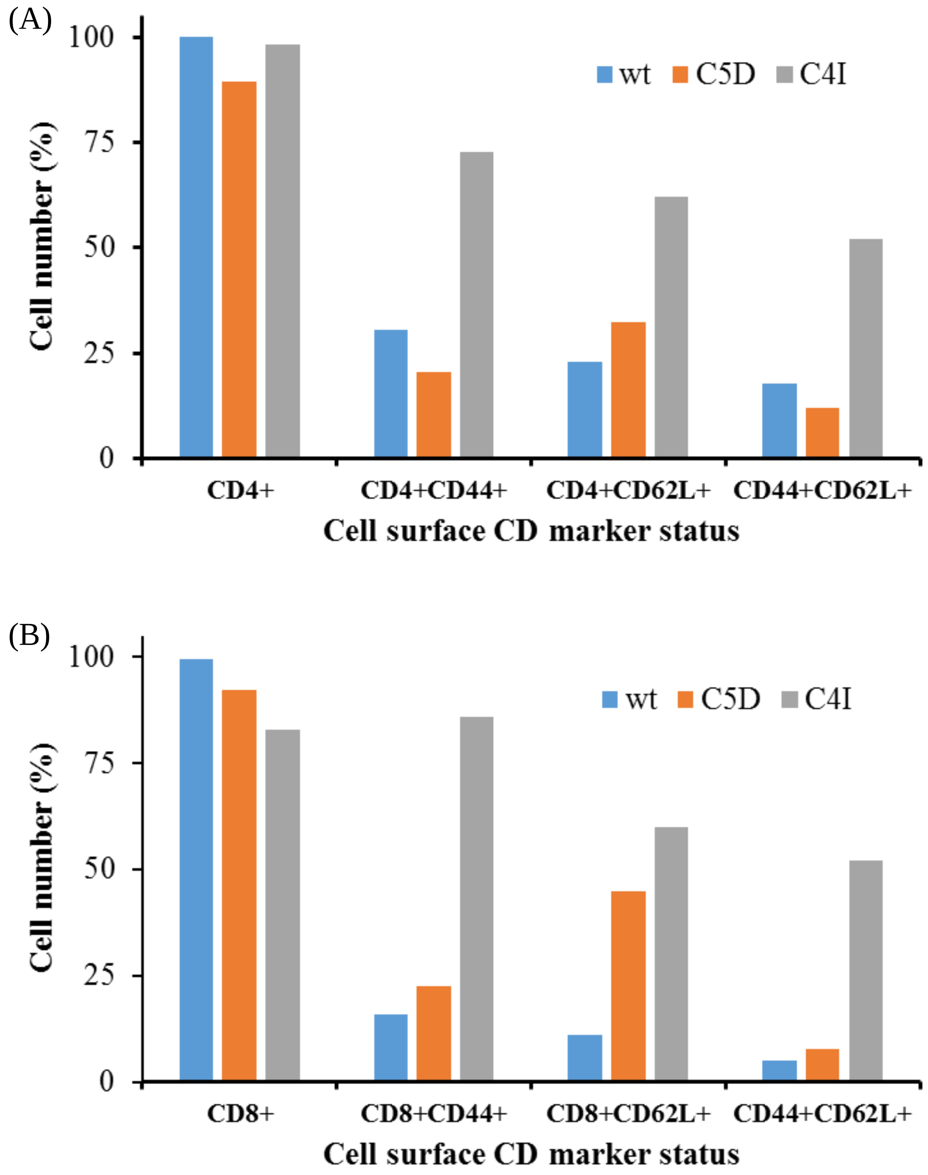
(A)
Cell number (%)
100
75
50
25
0
wt
C5D
C4I
CD4+
CD4+CD44+
CD4+CD62L+
CD44+CD62L+
Cell surface CD marker status
(B)
Cell number (%)
100
75
50
25
0
wt
C5D
C4I
CD8+
CD8+CD44+
CD8+CD62L+
CD44+CD62L+
Cell surface CD marker status
